# Correlation of Gut Microbiota, Vitamin D Status, and Pulmonary Function Tests in Children With Cystic Fibrosis

**DOI:** 10.3389/fnut.2022.884104

**Published:** 2022-06-09

**Authors:** Hadeel Albedewi, Iman Bindayel, Ahmed Albarrag, Hanaa Banjar

**Affiliations:** ^1^Department of Community Health Sciences, College of Applied Medical Sciences, King Saud University, Riyadh, Saudi Arabia; ^2^Department of Pathology, College of Medicine, King Saud University, Riyadh, Saudi Arabia; ^3^Department of Pediatrics, King Faisal Specialist Hospital and Research Center, Riyadh, Saudi Arabia

**Keywords:** cystic fibrosis, 25-hydroxyvitamin D, gut microbiota, real-time PCR, pulmonary function tests

## Abstract

**Background:**

Children with cystic fibrosis (CF) are expected to have suboptimal serum vitamin D status and altered gut microbiota. The altered gut microbiota is hypothesized to have a pro-inflammatory effect that further complicates the existing respiratory inflammation. Emerging evidence suggests an association between vitamin D and gut microbiota. The aim of this study was to assess the relationships between 25-hydroxyvitamin D [25(OH)D] status, pulmonary function, and fecal bacteria in children with CF.

**Methods:**

In this cross-sectional study, a total of 35 children with CF (8.7 ± 2.83 years) and 24 controls without CF (9 ± 2.7 years) were included in this study. Serum 25(OH)D status was measured using the Elecsys vitamin D total II assay. In the CF group, gut microbiota composition was assessed using real-time PCR analysis. Pulmonary function tests (PFTs) were measured using spirometry. Comparisons between the CF and non-CF controls were conducted using the independent sample *t*-test. In the CF group, one-way analysis of variance (ANOVA) was used to assess differences in PFTs and gut microbiota composition across the three vitamin D subgroups. The correlations between 25(OH)D status and PFTs, or gut microbiota composition, and PFTs with gut microbiota composition were analyzed using the Pearson's correlation coefficient test.

**Results:**

Children with CF had significantly lower serum 25(OH)D levels compared with children without CF (44.3 ± 22.4 vs. 59 ± 25.5, respectively, *P* = 0.026). Children with CF with optimal serum 25(OH)D level had significantly higher levels of *Bacteroidetes, Firmicutes*, and total bacteria (*P* = 0.007, *P* = 0.007, and *P* = 0.022, respectively). The level of *Firmicutes* was found to be significantly higher in mild forced expiratory volume in 1 s (FEV1) compared with moderate FEV1 (*P* = 0.032), whereas the level of the other bacteria species was comparable across FEV1 severity groups.

**Conclusion:**

Our findings may encourage studies that target and modify gut microbiota to potentially achieve better outcomes in terms of respiratory function in CF.

## Introduction

Cystic fibrosis (CF) is an autosomal recessive disorder caused by a mutation in CF transmembrane conductance regulator (CFTR) gene (1). There are several manifestations of CF, including chronic lung inflammation and repeated infections, and gastrointestinal abnormalities such as exocrine pancreatic insufficiency and fat malabsorption that leads to malnutrition. These characteristics of the disease impose gastrointestinal microbiota alteration and vitamin D deficiency or insufficiency that is often observed in individuals with CF (2). Vitamin D deficiency or insufficiency is common in patients with CF due to pancreatic insufficiency, fat malabsorption, low vitamin D binding protein level, corticosteroid medication use, and inadequate exposure to sunlight (3, 4). In addition to maintaining skeletal strength and bone mineralization, vitamin D has the ability to decrease colonization of the airways through stimulating antimicrobial peptides LL-37 that promote bronchial cell reaction to *Pseudomonas aeruginosa*, a pathogen related to CF (5). Furthermore, vitamin D was found to be an inhibitor of airway pro-inflammatory cytokines indicated by a significant reduction in interleukin 17A and interleukin 23 levels after vitamin D supplementation (6). It has also been observed that polymorphisms in vitamin D binding protein correlate with 25(OH)D levels and decreased pulmonary function measured by forced expiratory volume in 1 s (FEV1) in patients with CF (7).

Individuals with CF are expected to have gastrointestinal microbiota alteration due to changes in gastrointestinal mucus, pH, fat malabsorption, and antibiotic use (8). Moreover, the severity of CFTR mutation was found to be correlated with gut microbial imbalance, namely, an increase in pathogenic bacteria, such as *Escherichia coli* (*E*. *coli*), and a reduction in Bifidobacteria (9). The *E. coli* species produces pro-inflammatory mediators that initiate a low-grade inflammation environment, which may trigger systematic inflammation and eventually chronic lung inflammatory disease (8). Furthermore, a study showed a disease-specific pattern of gut microbiota alteration in CF, regardless of patients' age, antibiotic use, or pancreatic function (10). In fact, the intestine of patients with CF produces suboptimal bicarbonate levels, causing a lower pH level and thicker mucus and resulting in intestinal transit interruption (11). Therefore, the lower secretion of chloride and bicarbonate and higher uptake of water intestinal lumen causes dehydration, increases the lumen content acidity, and eventually compromises nutrient absorption (2). Individuals with CF manifest an impairment in mucus production and transport, creating a suitable environment for small intestine bacterial overgrowth. This was also related to a reduced diversity in commensal gut microbiota. As a result, the formerly mentioned factors act collectively in the progression of chronic low-grade inflammation and impaired immunity in the gut of individuals with CF (2). In fact, a study conducted on infants with CF has linked the alteration of the gut microbial community in CF to pulmonary exacerbation during the first year of life (12). Furthermore, a longitudinal study on 13 children with CF revealed a significant correlation between gut inhibiting bacteria and respiratory symptoms rather than respiratory inhabiting bacteria, namely; *Bacteroides* and *Bifidobacterium* (13). Another longitudinal study conducted on 7 CF neonates has demonstrated a matched variation and richness of several bacterial taxa inhabiting the gut and respiratory tract (14). Several bacterial species that were elevated in the gut were also found to be elevated in the respiratory tract. Interestingly, the gut microbiome colonization was found to precede that of the respiratory tract; for instance, the gut colonization of *Enterococcus* was followed by a cluster formation of these potentially pathogenic bacteria in the respiratory tract (14). The suggested pathways for the gut-lung axis include endothelium signal absorption by epithelial cells from local to distal sites, mesenteric lymphatic system, and local and long-reaching immune reactions (15, 16). Intestinal epithelial cells are directly stimulated by gut microbiota, which regulates immune cell release. Inflammation is linked to bacterial translocation, which involves the migration of microbes or their metabolites from the gastrointestinal system to the circulation across the mucosal barrier (17).

Recently, emerging evidence suggests an association between vitamin D and the composition of gut microbiota (18). A cross-sectional study has reported a negative correlation of vitamin D status with *Coprococcus* and Bifidobacteria in a healthy population (*r* = −0.22 and *r* = −0.27, *P* < 0.005, respectively). In addition, the study had shown the subgroup with higher dietary vitamin D intake to have a significantly higher abundance of *Prevotella* and lower abundance of *Haemophilus* and *Veillonella* compared with the lower dietary vitamin D intake subgroups (19). In CF, supplementation of vitamin D3 (50,000 IU per week) for 3 months among 23 adults with CF resulted in a significant increase in *Lactococcus* and a decrease in *Veillonella* and *Erysipelotrichaceae* when compared with placebo (20). The pathways in which vitamin D modulates the gut microbiota composition include reducing intestinal inflammation, decreasing gastrointestinal permeability, increasing intestinal barrier function, and stimulating antimicrobial peptides (18).

Therefore, due to the low number of studies investigating the association between vitamin D and gut microbiota in CF (20, 21), there is a need for more studies that compare vitamin D level in children with CF and children without CF and then examine its association with gut residents and pulmonary function outcomes. Therefore, this study aimed to assess 25-hydroxyvitamin D [25(OH)D] levels in patients with CF and controls without CF, then measure the fecal abundance of five of the most relevant bacteria species in patients with CF, and investigate their association with 25(OH)D status. In addition, relationships between 25(OH)D status and pulmonary function and fecal bacteria and pulmonary function were examined in the children with CF.

## Materials and Methods

### Participant Recruitment and Ethics Statement

In this cross-sectional study, children with CF who were stable with no acute pulmonary exacerbation, gastrointestinal symptoms, or antibiotic use for at least 2 weeks were recruited from the Cystic Fibrosis Clinic at King Faisal Specialist Hospital and Research Center (KFSH&RC), Riyadh, Saudi Arabia, during their regular clinical visits between July 2019 and December 2019. Moreover, children without CF were recruited from a pediatric clinic and classified as the non-CF control group. Parents were contacted before their appointments by their nurse coordinator to inform them about the study. Patients were screened by their pediatrician for inclusion and exclusion criteria. Anthropometrical measurements were performed by a nurse to assess for growth and malnutrition, and then pulmonary function testing was performed by a respiratory therapist. Blood samples routinely collected were used to assess serum 25(OH)D levels. Parents were then interviewed by the dietician to estimate their dietary intake of vitamin D. The protocol was approved by the institutional review board of KFSH&RC in the Office of Research Affairs at the Research Advisory Committee (RAC) with the project number RAC# 2191068. Written informed consent was obtained from all parents or caregivers of involved study children. All clinical investigations were performed in accordance with the Declaration of Helsinki. The inclusion criteria were as follows: (1) CF diagnosis based on clinical symptoms, high sweat chloride testing (>60 mmol/L), and CFTR mutational analysis, (2) stable condition (assessed by the pediatrician), and (3) age >5 years. The exclusion criteria were as follows: (1) antibiotic use within 3 weeks prior to fecal sample collection and (2) intellectual disability that affects the ability to perform pulmonary function test (PFT) accurately.

### Anthropometric Measurements

Anthropometric measurements including weight, height, and body mass index (BMI) were obtained from study subjects during their clinical visits by a registered nurse. BMI *z*-score and BMI percentile were calculated using Center for Disease Control (CDC) growth charts. The CDC cutoffs for BMI-for-age were used as follows: <5^th^ percentile is considered underweight, 5^th^ to <85^th^ percentile is healthy weight, 85^th^ to <95^th^ percentile is overweight, and >95^th^ percentile is obese (22).

### Total 25(OH)D Status

The measurement of total serum 25(OH)D levels was conducted at the laboratory of KFSH&RC using the Elecsys vitamin D total II assay on Cobas 801 immunoassay auto-analyzer system (Roche Diagnostics GmbA, Mannheim, Germany). The cutoff point for vitamin D deficiency and insufficiency was obtained from the Institute of Medicine where a 25(OH)D level of <30 nmol/L is considered vitamin D deficiency, a level between 30 and 50 nmol/L is considered vitamin D insufficiency, and the optimal vitamin D level was set at >50 nmol/L (23).

### Dietary Vitamin D Intake

The assessment of dietary vitamin D intake among study subjects was conducted through a validated food frequency questionnaire (FFQ) specific for calcium and vitamin D obtained from a previous study conducted on Saudi children (24). The amounts of vitamin D in each food item included in the questionnaire were adapted from the U.S. Department of Agriculture food composition database (25).

### Pulmonary Function Tests

Pulmonary function tests, including FEV1, forced vital capacity (FVC), FEV1/FVC ratio, and forced expiratory flow at 25 and 75% of the pulmonary volume (FEF_25−75%_), were performed using spirometry. All PFTs were expressed as percentages of predicted values. The cutoff points for the severity of airflow obstruction based on the percentage of the predicted value of FEV1 were as follows: FEV1% predicted >80% was considered as mild lung disease, while FEV1% predicted between 50% and 80% was moderate lung disease, and FEV1% predicted <50% was considered as severe lung disease (26).

### Fecal Microbiota Analysis

#### Collection and Storage

A freshly voided stool sample from each participant in the CF group was collected by their parents or caregivers in sterilized sample containers and kept at 4°C for a maximum of 3 h. Later, two aliquots weighing 250 mg were obtained from each fecal sample and then stored at −80°C for DNA extraction.

#### DNA Extraction

DNA extraction was performed on a single aliquot for each sample using the commercially available kit (QIAamp DNA stool mini kit; Qiagen, Hilden, Germany) following the manufacturer's instructions. The concentration and purity of extracted DNA were measured using the Nanodrop 2000 Spectrophotometer (Thermo Fisher Scientific, MA, USA) at 260 nm and 260/280 ratio, respectively. Extracted DNA samples were adjusted to a concentration of 10 ng/μl and stored at −20°C until they are analyzed for bacterial quantification by real-time PCR.

#### Standard Curve

Positive controls were used to construct a standard curve for total and target bacteria. Bacterial strains used as positive controls are shown in Table 1. Genomic DNA for each bacterial strain was extracted from pure cultures using the QIAamp DNA mini kit (Qiagen, Hilden, Germany) following the manufacturer's instructions. To generate the standard curves, we used ten-fold serial dilutions of genomic DNA extracted from pure cultures with known concentrations ranging from 1 × 10^8^ to 1 × 10^2^ colony-forming units per milliliter (CFU/ml) of each reference bacterium. We performed real-time PCR reactions and plotted the CT values against the log concentration of the reference bacterium copy number to create a linear regression.

**Table 1:** The primers and positive control used in this study.

**Primer**	**Sequence**	**Target**	**Positive control**	**References**
Bact934F	GGARCATGTGGTTTATTCGATGAT	*Bacteroidetes*	*B. fragilis*	(27)
Bact1060R	AGCTGACGACAACCATGCAG			
Eco1457F	CATTGACGTTACCCGCAGAAGAAGC	*Enterobacteriaceae*	*E. coli*	(28)
Eco1652R	CTCTACGAGACTCAAGCTTGC			
F-bifido	CGCGTCYGGTGTGAAAG	*Bifidobacterium spp*.	*B. longum*	(29)
R-bifido	CCCCACATCCAGCATCCA			
Firm934F	GGAGYATGTGGTTTAATTCGAAGCA	*Firmicutes*	*C. perfringens*	(27)
Firm1060R	AGCTGACGACAACCATGCAC			
Lacto-F	AGCAGTAGGGAATCTTCCA	*Lactobacillus spp*.	*L. rhamnosus*	(30)
Lacto-R	CACCGCTACACATGGAG			
UnivF	TCCTACGGGAGGCAGCAGT	Total	*L. rhamnosus*	(30)
UnivR	GGACTACCAGGGTATCTATCCTGTT			

#### Real-Time PCR

PCR primers were selected to determine the abundance of the gut microbiota strains of interest (Table 1). Real-time PCR was performed in a 50 μl reaction run in triplicate. Each reaction consists of 25 μl QuantiTect SYBR Green PCR Master Mix (Qiagen, Hilden, Germany), 7.5 μl of each forward and reverse primer, and 10 μl of DNA. Amplification was performed using Rotor-Gene Q (Qiagen, Hilden, Germany) starting with denaturation at 95°C for 15 min, followed by 40 cycles of denaturation at 94°C for 15 s, annealing for 30 s, and extension at 72°C for 40 s. The annealing temperature was 61.5°C for *E. coli*, 59°C for Bifidobacteria, 60.44°C for *Firmicutes*, and 60°C for *Bacteroidetes, Lactobacillus*, and total bacteria. The genome copy number of each reaction was calculated using the standard curves. Additionally, to calculate the total genome copy number per gram of wet stool, we used the equation (QM × C × DV)/(S × V) adapted from Metzler-Zebeli et al. (31) where QM is the copy number quantitative mean, C is the sample DNA concentration, DV is the dilution volume of extracted DNA, S is the amount of DNA used in the reaction, and V is the initial sample weight that was used for DNA extraction (31).

### Statistical Analysis

All data analyses were performed using Statistical Package for Social Science Statistics IBM SPSS for Macintosh, version 25.0 (IBM Corp., Armonk, N.Y., USA). Descriptive data are expressed as mean and standard deviation. Comparisons between the CF and non-CF control were conducted using the independent sample *t*-test. In the CF group, one-way analysis of variance (ANOVA) was used to assess differences in PFTs and gut microbiota composition across the three vitamin D subgroups (i.e., deficiency, insufficiency, and optimal). The correlations between 25(OH)D status and PFTs, or gut microbiota composition, and PFTs with gut microbiota composition were analyzed using the Pearson's correlation coefficient (two-tailed) test. A *P*-value <0.05 was considered significant in all tests.

## Results

In this cross-sectional study, a total of 35 children (16 males; 19 females, mean age, 8.7 ± 2.83 years) with CF and 24 non-CF control (14 males; 10 females, mean age, 9 ± 2.7 years) were included. The general characteristics of participants are shown in Table 2. The CF group had significantly lower BMI and BMI *z*-score compared with the non-CF group (*P* = 0.002, *P* = 0.016, respectively). In addition, there was a significant difference in regional distribution between the groups, with the majority of the CF children (54%) living in the Eastern region of Saudi Arabia (*P* = 0.001) (Table 2).

**Table 2:** Study participant characteristics.

**Variable**	**CF**	**Non-CF**	***P*-value**
	***N* = 35**	***N* = 24**	
Age, years, mean (SD)	8.7 (2.83)	9 (2.7)	0.601
Sex (male/female)	16/19	14/10	0.341
Region, *n* (%)			0.001
Central	7 (20)	15 (63)	
East	19 (54)	2 (8)	
West	1 (3)	1 (4)	
North	5 (14)	2 (8)	
South	3 (9)	4 (17)	
Height, cm, mean (SD)	124 (15.57)	123 (18.3)	0.805
Weight, kg, mean (SD)	22.9 (7.11)	27 (11.1)	0.107
BMI, kg/m^2^, mean (SD)	14.6 (1.4)	17.3 (3.8)	0.002
BMI z-score, mean (SD)	−1.3 (1.4)	−0.23 (1.8)	0.016
BMI-for-age, *n* (%)			0.148
Underweight	13 (38)	6 (25)	
Normal	20 (59)	14 (58)	
Overweight	1 (3)	4 (17)	
Serum 25(OH)D (nmol/L), mean (SD)	44.3 (22.4)	59 (25.5)	0.026
Serum 25(OH)D status (n/%)			0.083
Deficiency	8 (24.2)	1 (4)	
Insufficiency	14 (42.4)	10 (42)	
Optimal	11 (33.3)	13 (54)	
Vitamin D (supplements) (IU/day), mean (SD)	2,912 (1,083)	–	–
Vitamin D dietary intake (FFQ) (IU/day), mean (SD)	365 (141)	–	–
Vitamin D total intake (IU/day), mean (SD)	3,090 (1,288)	–	–
Pulmonary function testing			
Fev1 (%), mean (SD)	71 (27)	–	–
FVC (%), mean (SD)	72 (27)	–	–
FEV1/FVC (%), mean (SD)	97 (13)	–	–
FEF _25−75_ (%), mean (SD)	74 (35)	–	–

*The independent sample t-test was used to determine a significant difference in continuous variables between the CF and non-CF groups. Pearson chi-square test was used to determine a significant difference in the categorical variables between the CF and non-CF groups.*

*25(OH)D, 25-hydroxyvitamin D; SD, standard deviation; CD, cystic fibrosis*.

### Vitamin D in Serum and Dietary Intake

The total serum 25(OH)D level of CF children was significantly lower compared with non-CF children (independent sample *t*-test, *P* = 0.026, Table 2). There was no significant difference in age, sex, and BMI across the vitamin D subgroups in both children groups. The median estimated dietary intake of vitamin D and calcium for the CF group was 60% and 69.8% of the Recommended Dietary Allowance, respectively (Table 2).

### Pulmonary Function Tests

The PFTs were not different among the vitamin D subgroups in children with CF. Similarly, there was no significant correlation between PFT and vitamin D serum level. However, there was a significant positive correlation between BMI *z*-score and each one of the PFTs: FEV1, FVC, and FEF _25−75%_ (*r* = 0.515, *P* = 0.002; *r* = 0.472, *P* = 0.005; and *r* = 0.358, *P* = 0.041, respectively, data not shown).

### Gut Microbiota

**Figure 1:**
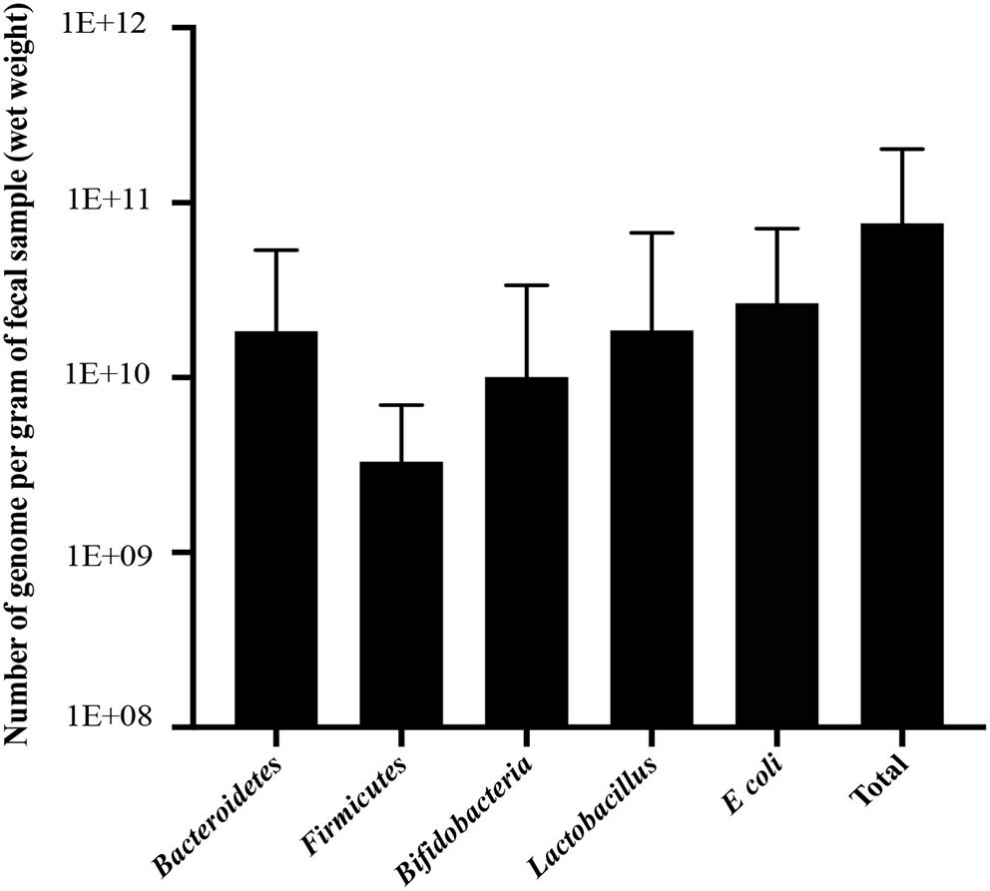
Number of genome copies per 1 g of fecal sample (wet weight) for *Bacteroidetes, Firmicutes*, Bifidobacteria*, Lactobacillus, E. coli*, and total bacteria in children with CF. Data are presented as mean and standard deviation (SD).

**Figure 2:**
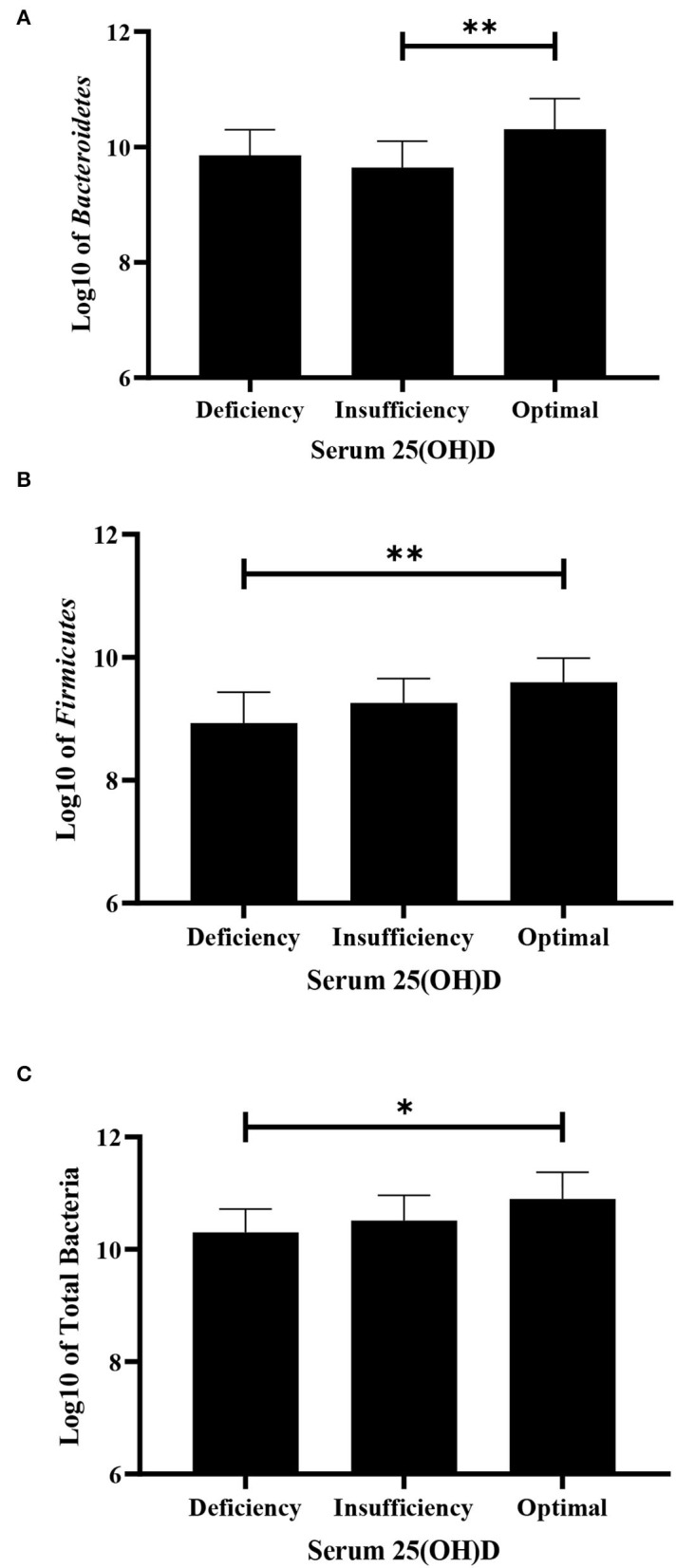
Difference in fecal bacteria strains among deficiency, insufficiency, and optimal serum vitamin D in children with CF using ANOVA. **(A)** *Bacteroidetes*. **(B)** *Firmicutes*. **(C)** Total bacteria in children with CF. Values are shown as mean and SD of log-transformed genome copies/g of fecal sample. *P < 0.05; **P < 0.005.

**Figure 3:**
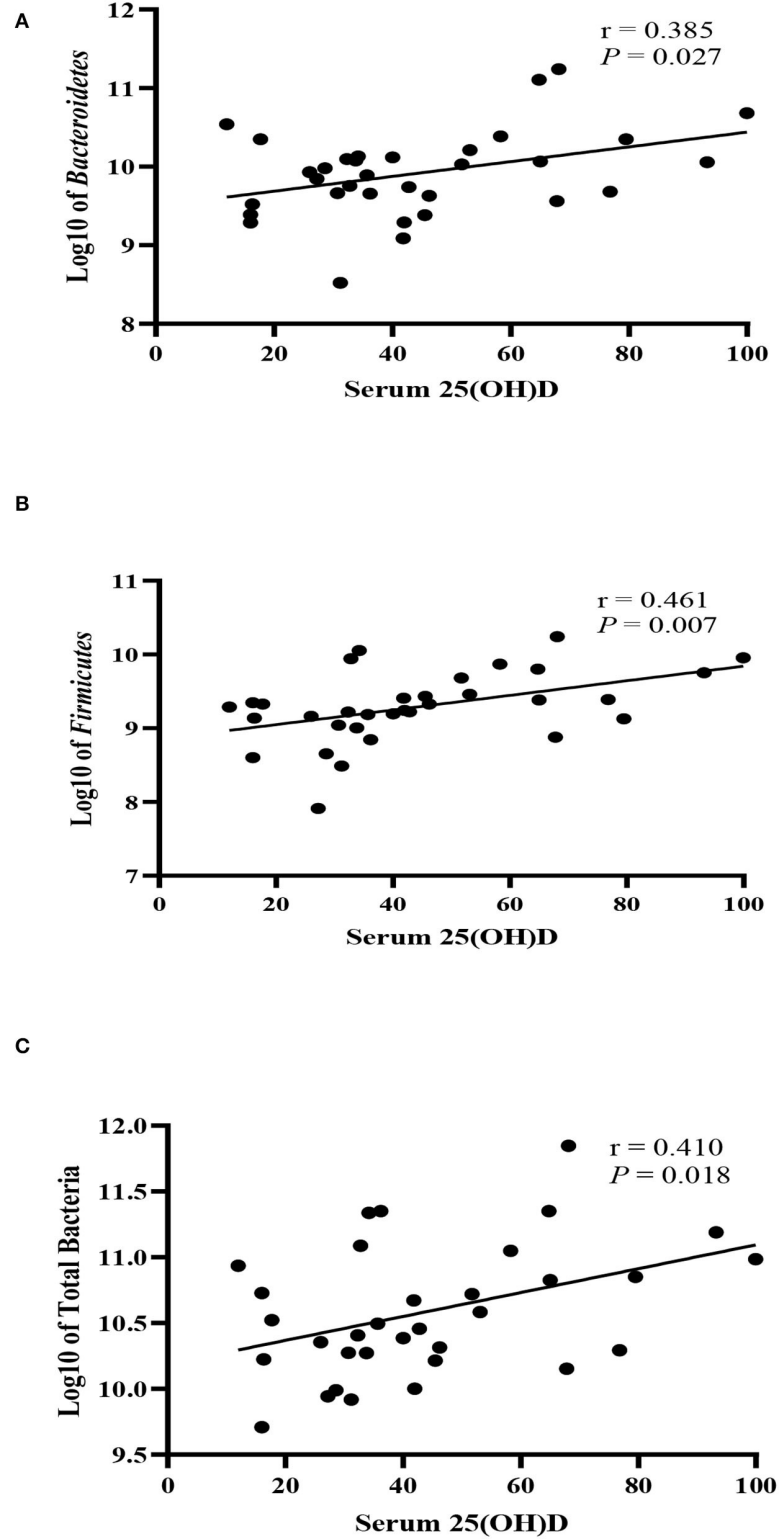
Correlation between fecal bacteria strains and serum 25-hydroxyvitamin D level (nmol/L) in children with CF. **(A)** *Bacteroidetes*, **(B)** *Firmicutes*, **(C)** Total bacteria. *r* denotes Pearson's correlation coefficient.

The bacterial copy number in feces for target and total bacteria is shown in Figure 1. Data are shown as the log10 of the number of genome copies/g of a fecal sample. Children with CF with optimal serum vitamin D had significantly higher levels of *Bacteroidetes, Firmicutes*, and total bacteria (*P* = 0.007, *P* = 0.007, and *P* = 0.022, respectively, Figure 2). Correspondingly, *Bacteroidetes, Firmicutes*, and total bacteria were found to be positively correlated with serum 25(OH)D level (Pearson's correlation, *r* = 0.385 *P* = 0.027; *r* = 0.461, *P* = 0.007; and *r* = 0.410, *P* = 0.018, respectively, Figure 3). However, there was no difference in Bifidobacteria, *Lactobacillus spp., E. coli*, and *Firmicutes/Bacteroidetes* ratio across vitamin D groups in children with CF (*P* > 0.05). Yet, there was a significant negative correlation between the serum 25(OH)D status and the ratio of *E. coli* to total bacteria (*r*_s_ = −0.382, *P* = 0.028).

**Figure 4:**
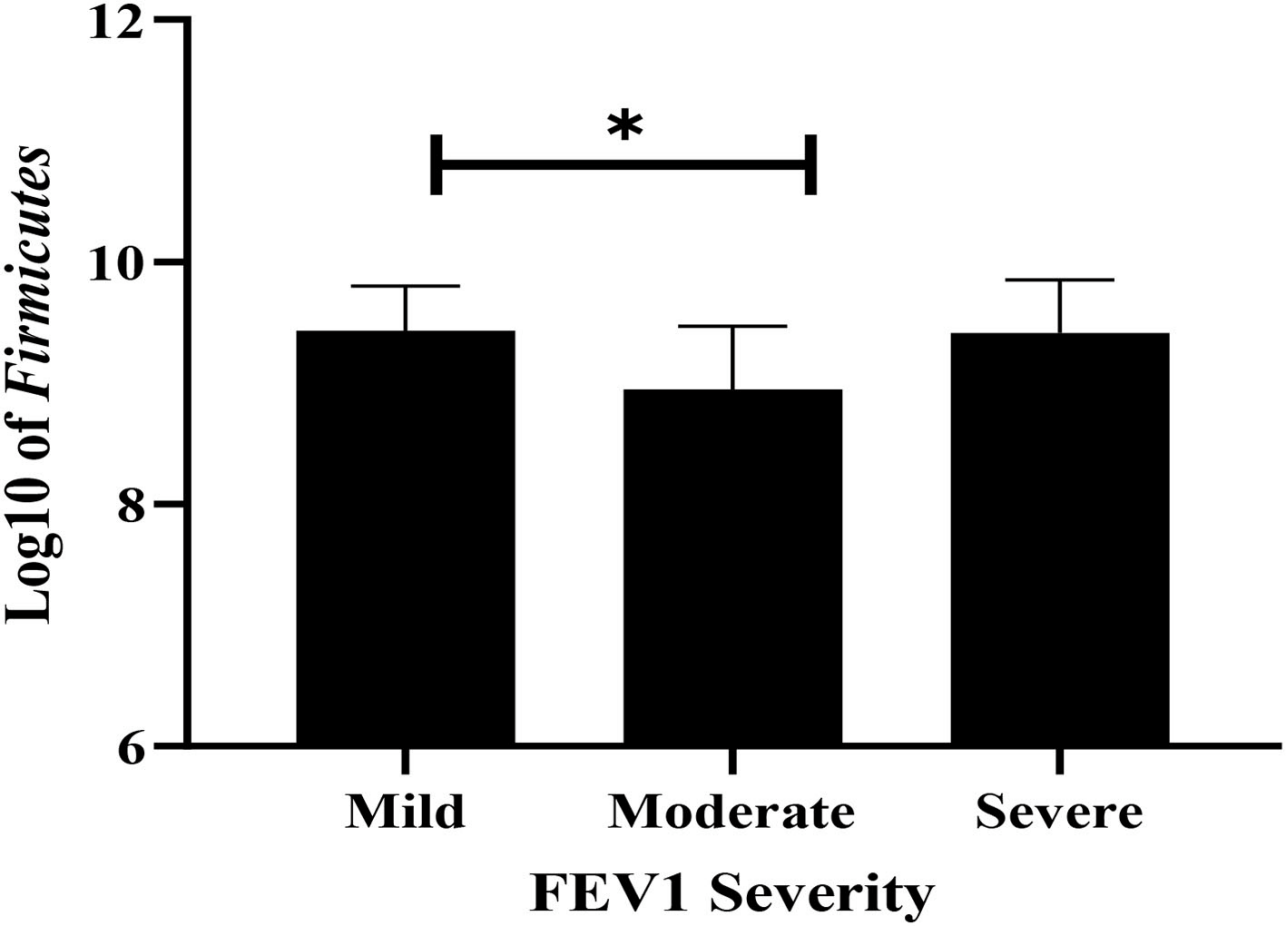
The difference of *Firmicutes* among mild, moderate, and severe airway obstruction using ANOVA. Values are shown as mean and SD of log-transformed genome copies/g of fecal sample. * *P* < 0.05.

Fecal bacterial strains were compared across the subgroups divided according to the severity of the pulmonary malfunction using one-way ANOVA. The *Firmicutes* level was significantly higher in mild FEV1 compared with the moderate FEV1 group (*P* = 0.032, Figure 4), whereas *Bacteroidetes, Lactobacillus, E. coli*, Bifidobacteria, and total bacteria levels did not differ across groups.

## Discussion

This study was conducted to investigate vitamin D hypovitaminosis and its association with PFTs and gut microbiota in children with CF. Our study showed a significantly lower serum 25(OH)D level in children with CF than in children without CF, and this is in accordance with previous observations suggesting a lower serum vitamin D level in children with CF (32). Reasons for the development of suboptimal vitamin D status in children with CF were attributed to the nature of CF disease that results in pancreatic insufficiency, reduced vitamin D binding protein levels, and fat malabsorption, therefore, vitamin D malabsorption (3, 4). Moreover, the clinical management of CF often necessitates the use of corticosteroids that may increase the expression of vitamin D catabolizing enzyme CYP24, in addition to some antibiotics that cause photosensitivity and lead to lower exposure to sunlight (3, 4). The mean BMI-for-age for children with CF in our sample was between the 5^th^ and 15^th^ percentile, with 40% of the sample being at <5^th^ percentile, which indicates that they either are nutritionally at risk or have a nutritional failure (33). Children with CF are known to have lower than normal body weight due to energy loss that is caused by maldigestion and malabsorption, increased energy expenditure, and decreased energy intake, resulting from anorexia and gastrointestinal symptoms (34). In fact, our study has shown significantly lower BMI and BMI *z*-score in children with CF compared with children without CF.

Previous studies had shown a significant positive association between vitamin D status and PFTs (35–37); however, this study was unable to demonstrate a significant association between 25(OH)D and PFTs. A possible explanation might be the fact that the majority of our sample (69%) had mild to moderate airflow obstruction with no pulmonary exacerbations. In fact, few previous studies were unable to demonstrate any significant association between vitamin D status and PFTs; rather, a significant association with pulmonary exacerbation was demonstrated (38, 39).

It was hypothesized that gut microbiota composition is positively correlated with serum vitamin D status. In this study, gut microbiota composition was found to be significantly different across serum vitamin D subgroups. Particularly, the levels of *Bacteroidetes, Firmicutes*, and total bacteria were significantly higher in the optimal vitamin D group. These results are consistent with those obtained from Kanhere et al. (20), who found that the level of *Bacteroidia*, a bacterial class belonging to *Bacteroidetes*, was higher in adults with optimal vitamin D. Another important finding in this study was the negative correlation between vitamin D status and *E. coli*. A significant association was found between suboptimal vitamin D levels and a higher abundance of *Gammaproteobacteria*, a class of bacteria that includes *E. coli* (40). This link between vitamin D status and microbial colonization can be explained by the significant role vitamin D plays in modulating the immune system in the intestine (41). The absence of the anticipated association between beneficial species such as Bifidobacteria and *Lactobacillus* spp. and vitamin D level in this study is perhaps related to the redundant effect of vitamin D at its optimal level to modify the existing dysbiosis related to the pro-inflammatory environment in children with CF. In fact, this may highlight the need to increase the currently prescribed regimen for vitamin D in children with CF to reach visible changes in gut dysbiosis. Clinical studies that assess the effect of vitamin D supplementation on gut dysbiosis in children with CF are warranted.

With respect to the association between gastrointestinal bacterial species and PFTs, our study showed higher levels of *Firmicutes* in children with mild airflow obstruction compared with those with moderate obstruction. Similarly, a previous study showed a significantly higher level of *Firmicutes* in children with CF with mild lung disease than those with moderate lung disease (42). This can be explained by the role *Firmicutes* play in producing short-chain fatty acids (SCFAs), particularly butyrate, which has been shown to downregulate pro-inflammatory factors, such as IL-6 and IL-12 (43). SCFAs produced by *Bacteroides, Bifidobacterium, Fecalibacterium*, and *Enterobacteria* are also known for promoting the release of anti-inflammatory cytokines (IL-10 and IL-21) and the inhibition of lipopolysaccharide (LPS)-induced pro-inflammatory cytokines (44, 45). As a result, SCFA plays a major role in the modulation of intestinal regulatory T cells which are essential in which they maintain the host immunological homeostasis (44). In CF, the increase in pro-inflammatory factors, attributed to CFTR deficiency in the epithelial cells of the airways, has been linked to the exacerbation of lung function (46, 47).

We acknowledge that our study has certain limitations. The major limitation of this study is the absence of gut microbiota composition assessment of children without CF, due to lack of access to their samples. Furthermore, since the study excluded unstable children with CF, it was not possible to evaluate the effect of vitamin D status and gut microbiota composition on pulmonary exacerbations. Finally, this study was cross-sectional in nature, which neglects the potential prospective impact. Despite these limitations, this study has some strengths as it is a nutrition-inclusive study that considered nutritional status factors such as BMI and nutritional intake, while investigating the associations among vitamin D status, PFTs, and gut microbiota composition in children with CF. To the best of our knowledge, this is the first study to assess gut microbiota composition in children with CF in Saudi Arabia. With only a few exceptions, blood and stool samples were collected on the same day as spirometry was performed. In addition, the use of antibiotics was eliminated for at least 3 weeks prior to data collection to minimize its influence on gut microbiota composition.

## Conclusion

The most significant finding to emerge from this study is the difference in serum 25(OH)D levels between the CF and non-CF groups. Another important finding is the significant difference in gut microbiota composition in relation to vitamin D status and PFTs in children with CF. This study identified two phyla (*Firmicutes* and *Bacteroidetes*) to be significantly associated with optimal serum vitamin D levels and one species that has a negative correlation with serum vitamin D, which is *E. coli*. Moreover, this study has provided a deeper insight into the relationship between gut microbiota and pulmonary outcome as it demonstrated a positive correlation of PFTs with *Firmicutes* and the ratio of *Firmicutes* to *Bacteroidetes*. Our findings strengthen the idea of modifying gut microbiota to achieve better outcomes in CF in terms of pulmonary function. Future studies need to be conducted to investigate the benefit of probiotics use as a part of clinical management for CF and measuring its impact on clinical outcomes, such as pulmonary function and pulmonary exacerbations, and vitamin D status.

## Data Availability Statement

The original contributions presented in the study are included in the article/supplementary material, further inquiries can be directed to the corresponding author/s.

## Ethics Statement

The studies involving human participants were reviewed and approved by Research Affairs at the Research Advisory Committee (RAC) at King Faisal Specialist Hospital and Research Center (RAC# 2191068). Written informed consent to participate in this study was provided by the participants' legal guardian/next of kin.

## Author Contributions

HA and IB conceptualized and designed this study, statistically analyzed the data, prepared the manuscript, and revised its final version. HA and HB screened and recruited patients and collected the study outcomes. HA performed and supervised sample collection and analysis. HA and AA designed and conducted gut microbiota analysis. IB supervised the implementation of all steps in this research. All authors contributed to the article and approved the submitted version.

## Funding

This study was supported by a grant from the Research Center of the Female Scientific and Medical Colleges, Deanship of Scientific Research, King Saud University.

## Conflict of Interest

The authors declare that the research was conducted in the absence of any commercial or financial relationships that could be construed as a potential conflict of interest.

## Publisher's Note

All claims expressed in this article are solely those of the authors and do not necessarily represent those of their affiliated organizations, or those of the publisher, the editors and the reviewers. Any product that may be evaluated in this article, or claim that may be made by its manufacturer, is not guaranteed or endorsed by the publisher.

## Abbreviations

25(OH)D: 25-hydroxyvitamin D
ANOVA: analysis of variance
BMI: body mass index
CDC: Center for Disease Control
CF: cystic fibrosis
CFTR: CF transmembrane conductance regulator
FEV1: forced expiratory volume in 1 s
FFQ: food frequency questionnaire
FVC: forced vital capacity
KFSH&RC: King Faisal Specialist Hospital and Research Center
PFT: pulmonary function tests
RAC: Research Advisory Committee.

## References

[1] Elborn JS. Cystic fibrosis. Lancet. (2016) 388:2519–31. 10.1016/s0140-6736(16)00576-6 27140670

[2] Bowen S-J Hull J. The basic science of cystic fibrosis. Paediatr Child Health. (2015) 25:159–64. 10.1016/j.paed.2014.12.008

[3] Chesdachai S Tangpricha V. Treatment of vitamin D deficiency in cystic fibrosis. J Steroid Biochem Mol Biol. (2016) 164:36–9. 10.1016/j.jsbmb.2015.09.013 26365559 PMC4786457

[4] Herscovitch K Dauletbaev N Lands LC. Vitamin D as an anti-microbial and anti-inflammatory therapy for cystic fibrosis. Paediatr Respir Rev. (2014) 15:154–62. 10.1016/j.prrv.2013.11.002 24332502

[5] Kempker JA Tangpricha V Ziegler TR Martin GS. Vitamin D in sepsis: from basic science to clinical impact. Crit Care. (2012) 16:316. 10.1186/cc11252 22809263 PMC3580673

[6] Olszowiec-Chlebna M Koniarek-Maniecka A Brzozowska A Blauz A Rychlik B Stelmach I. Vitamin D inhibits pro-inflammatory cytokines in the airways of cystic fibrosis patients infected by Pseudomonas aeruginosa- pilot study. Ital J Pediatr. (2019) 45:41. 10.1186/s13052-019-0634-x 30922377 PMC6440129

[7] Speeckaert MM Wehlou C Vandewalle S Taes YE Robberecht E Delanghe JR. Vitamin D binding protein, a new nutritional marker in cystic fibrosis patients. Clin Chem Lab Med. (2008) 46:365–70. 10.1515/CCLM.2008.084 18303991

[8] Rogers GB Narkewicz MR Hoffman LR. The CF gastrointestinal microbiome: Structure and clinical impact. Pediatr Pulmonol. (2016) 51:S35–44. 10.1002/ppul.23544 27662102 PMC5303757

[9] Schippa S Iebba V Santangelo F Gagliardi A De Biase RV Stamato A. Cystic fibrosis transmembrane conductance regulator (CFTR) allelic variants relate to shifts in faecal microbiota of cystic fibrosis patients. PLoS ONE. (2013) 8:e61176. 10.1371/journal.pone.0061176 23613805 PMC3629184

[10] Vernocchi P Del Chierico F Russo A Majo F Rossitto M Valerio M. Gut microbiota signatures in cystic fibrosis: loss of host CFTR function drives the microbiota enterophenotype. PLoS ONE. (2018) 13:e0208171. 10.1371/journal.pone.0208171 30521551 PMC6283533

[11] Ooi CY Durie PR. Cystic fibrosis from the gastroenterologist's perspective. Nat Rev Gastroenterol Hepatol. (2016) 13:175–85. 10.1038/nrgastro.2015.226 26790364

[12] Antosca KM Chernikova DA Price CE Ruoff KL Li K Guill MF. Altered stool microbiota of infants with cystic fibrosis shows a reduction in genera associated with immune programming from birth. J Bacteriol. (2019) 201:e00274-19. 10.1128/JB.00274-19 PMC6657602 31209076

[13] Hoen AG Li J Moulton LA O'Toole GA Housman ML Koestler DC. Associations between gut microbial colonization in early life and respiratory outcomes in cystic fibrosis. J Pediatrics. (2015) 167:138–47e131–133. 10.1016/j.jpeds.2015.02.049 PMC4674690 25818499

[14] Madan JC Koestler DC Stanton BA Davidson L Moulton LA Housman ML. Serial analysis of the gut and respiratory microbiome in cystic fibrosis in infancy: interaction between intestinal and respiratory tracts and impact of nutritional exposures. mBio. (2012) 3:e00251-12. 10.1128/mBio.00251-12 PMC3428694 22911969

[15] Barcik W Boutin RCT Sokolowska M Finlay BB. The role of lung and gut microbiota in the pathology of asthma. Immunity. (2020) 52:241–55. 10.1016/j.immuni.2020.01.007 32075727 PMC7128389

[16] Enaud R Prevel R Ciarlo E Beaufils F Wieers G Guery B. The gut-lung axis in health and respiratory diseases: a place for inter-organ and inter-kingdom crosstalks. Front Cell Infect Microbiol. (2020) 10:9. 10.3389/fcimb.2020.00009 32140452 PMC7042389

[17] Zhao Y Liu Y Li S Peng Z Liu X Chen J. Role of lung and gut microbiota on lung cancer pathogenesis. J Cancer Res Clin Oncol. (2021) 147:2177–86. 10.1007/s00432-021-03644-0 34018055 PMC8236441

[18] Sun J. VDR/vitamin D receptor regulates autophagic activity through ATG16L1. Autophagy. (2016) 12:1057–8. 10.1080/15548627.2015.1072670 26218741 PMC4922437

[19] Luthold RV Fernandes GR Franco-de-Moraes AC Folchetti LG Ferreira SR. Gut microbiota interactions with the immunomodulatory role of vitamin D in normal individuals. Metabolism. (2017) 69:76–86. 10.1016/j.metabol.2017.01.007 28285654

[20] Kanhere M He J Chassaing B Ziegler TR Alvarez JA Ivie EA. Bolus weekly vitamin D3 supplementation impacts gut and airway microbiota in adults with cystic fibrosis: a double-blind, randomized, placebo-controlled clinical trial. J Clin Endocrinol Metab. (2018) 103:564–74. 10.1210/jc.2017-01983 29161417 PMC5800825

[21] Li L Krause L Somerset S. Associations between micronutrient intakes and gut microbiota in a group of adults with cystic fibrosis. Clin Nutr. (2017) 36:1097–104. 10.1016/j.clnu.2016.06.029 27595636

[22] Barlow SE. Expert committee recommendations regarding the prevention, assessment, and treatment of child and adolescent overweight and obesity: summary report. Pediatrics. (2007) 120 Suppl 4:S164–192. 10.1542/peds.2007-2329C 18055651

[23] Ross A Taylor C Yaktine A. Committee to Review Dietary Reference Intakes for Vitamin D and Calcium. Overview of Vitamin D. Washington DC: The National Academies Press (2011). p. 345–402 21796828

[24] Al-Musharaf S Al-Othman A Al-Daghri NM Krishnaswamy S Yusuf DS Alkharfy KM. Vitamin D deficiency and calcium intake in reference to increased body mass index in children and adolescents. Eur J Pediatr. (2012) 171:1081–6. 10.1007/s00431-012-1686-8 22311168

[25] US Department of Agriculture. Agricultural Research Service, Food Data Central. (2019). Available online at: https://fdc.nal.usda.gov/ (accessed July 03, 2020).

[26] Fabbri L Pauwels RA Hurd SS. Global strategy for the diagnosis, management, and prevention of chronic obstructive pulmonary disease: GOLD Executive Summary updated 2003. Int J Chronic Obstruct Pulm Dis. (2004) 1:105–41; discussion 103–104. 10.1081/COPD-120030163 16997745

[27] Guo X Xia X Tang R Zhou J Zhao H Wang K. Development of a real-time PCR method for Firmicutes and Bacteroidetes in faeces and its application to quantify intestinal population of obese and lean pigs. Lett Appl Microbiol. (2008) 47:367–73. 10.1111/j.1472-765X.2008.02408.x 19146523

[28] Bartosch S Fite A Macfarlane GT McMurdo ME. Characterization of bacterial communities in feces from healthy elderly volunteers and hospitalized elderly patients by using real-time PCR and effects of antibiotic treatment on the fecal microbiota. Appl Environ Microbiol. (2004) 70:3575–81. 10.1128/AEM.70.6.3575-3581.2004 15184159 PMC427772

[29] Delroisse JM Boulvin AL Parmentier I Dauphin RD Vandenbol M Portetelle D. Quantification of *Bifidobacterium* spp. and *Lactobacillus* spp in rat fecal samples by real-time PCR. Nat Microbiol. (2008) 163:663–70. 10.1016/j.micres.2006.09.004 19216105

[30] Wang L Christophersen CT Sorich MJ Gerber JP Angley MT Conlon MA. Low relative abundances of the mucolytic bacterium Akkermansia muciniphila and Bifidobacterium spp. in feces of children with autism. Appl Environ Microbiol. (2011) 77:6718–21. 10.1128/AEM.05212-11 21784919 PMC3187122

[31] Metzler-Zebeli BU Mann E Schmitz-Esser S Wagner M Ritzmann M Zebeli Q. Changing dietary calcium-phosphorus level and cereal source selectively alters abundance of bacteria and metabolites in the upper gastrointestinal tracts of weaned pigs. Appl Environ Microbiol. (2013) 79:7264–72. 10.1128/AEM.02691-13 24038702 PMC3837733

[32] Rovner AJ Stallings VA Schall JI Leonard MB Zemel BS. Vitamin D insufficiency in children, adolescents, and young adults with cystic fibrosis despite routine oral supplementation. Am J Clin Nutr. (2007) 86:1694–9. 10.1093/ajcn/86.5.1694 18065588

[33] Borowitz D Baker RD Stallings V. Consensus report on nutrition for pediatric patients with cystic fibrosis. J Pediatr Gastroenterol Nutr. (2002) 35:246–59. 10.1097/00005176-200209000-00004 12352509

[34] Slae M Wilschanski M. Prevention of malnutrition in cystic fibrosis. Curr Opin Pulm Med. (2019) 25:674–9. 10.1097/MCP.0000000000000629 31567328

[35] Loukou I Moustaki M Sardeli O Plyta M Douros K. Association of vitamin D status with lung function measurements in children and adolescents with cystic fibrosis. Pediatr Pulmonol. (2020) 55:1375–80. 10.1002/ppul.24460 31338968

[36] Norton L Page S Sheehan M Mazurak V Brunet-Wood K Larsen B. Prevalence of inadequate vitamin d status and associated factors in children with cystic fibrosis. Nutr Clin Pract. (2015) 30:111–6. 10.1177/0884533614562839 25550329

[37] Sexauer WP Hadeh A Ohman-Strickland PA Zanni RL Varlotta L Holsclaw D. Vitamin D deficiency is associated with pulmonary dysfunction in cystic fibrosis. J Cystic Fibrosis. (2015) 14:497–506. 10.1016/j.jcf.2014.12.006 25577127

[38] McCauley LA Thomas W Laguna TA Regelmann WE Moran A Polgreen LE. Vitamin D deficiency is associated with pulmonary exacerbations in children with cystic fibrosis. Ann Am Thorac Soc. (2014) 11:198–204. 10.1513/AnnalsATS.201208-068OC 24083951 PMC3972970

[39] Ongaratto R Rosa KMD Eloi JC Epifanio M Marostica P Pinto LA. Association between hypovitaminosis D and frequency of pulmonary exacerbations in children and adolescents with cystic fibrosis. Einstein. (2018) 16, eAO. 10.1590/s1679-45082018ao4143 PMC6063747 29694616

[40] Kanhere M Chassaing B Gewirtz AT Tangpricha V. Role of vitamin D on gut microbiota in cystic fibrosis. J Steroid Biochem Mol Biol. (2018) 175:82–7. 10.1016/j.jsbmb.2016.11.001 27818276 PMC5415426

[41] Christakos S Hewison M Gardner DG Wagner CL Sergeev IN Rutten E. Vitamin D: beyond bone. Ann N Y Acad Sci. (2013) 1287:45–58. 10.1111/nyas.12129 23682710 PMC3717170

[42] Burke DG Fouhy F Harrison MJ Rea MC Cotter PD O'Sullivan O. The altered gut microbiota in adults with cystic fibrosis. BMC Microbiol. (2017) 17:58. 10.1186/s12866-017-0968-8 28279152 PMC5345154

[43] Chang PV Hao L Offermanns S Medzhitov R. The microbial metabolite butyrate regulates intestinal macrophage function *via* histone deacetylase inhibition. Proc Nat Acad Sci. (2014) 111:2247–52. 10.1073/pnas.1322269111 24390544 PMC3926023

[44] Alsharairi N. A. (2020). The role of short-chain fatty acids in the interplay between a very low-calorie ketogenic diet and the infant gut microbiota and its therapeutic implications for reducing asthma. Int J Mol Sci. 21:24. 10.3390/ijms21249580 PMC7765661 33339172

[45] Shukla SD Budden KF Neal R Hansbro PM. Microbiome effects on immunity, health and disease in the lung. Clin Transl Immunology. (2017) 6:e133. 10.1038/cti.2017.6 28435675 PMC5382435

[46] Castaldo A Iacotucci P Carnovale V Cimino R Liguori R Comegna M. Salivary cytokines and airways disease severity in patients with cystic fibrosis. Diagnostics. (2020) 10:222. 10.3390/diagnostics10040222 32326546 PMC7235910

[47] Cohen-Cymberknoh M Kerem E Ferkol T Elizur A. Airway inflammation in cystic fibrosis: molecular mechanisms and clinical implications. Thorax. (2013) 68:1157–62. 10.1136/thoraxjnl-2013-203204 23704228

